# Three-dimensional spatial interpolation for chlorophyll-*a* and its application in the Bohai Sea

**DOI:** 10.1038/s41598-023-35123-6

**Published:** 2023-05-16

**Authors:** Zihan Zhao, Rushui Xiao, Junting Guo, Yuming Zhang, Shufang Zhang, Xianqing Lv, Honghua Shi

**Affiliations:** 1 Frontier Science Center for Deep Ocean Multispheres and Earth System (FDOMES) and Physical Oceanography Laboratory, Ocean University of China, Qingdao, China; 2 South China Institute of Environmental Sciences, Ministry of Ecology and Environment, Guangzhou, 510530 China; 3 National Marine Environment Monitoring Center, Dalian, 116023 China; 4 First Institute of Oceanography, Ministry of Natural Resources, Qingdao, 266061 China; 5 Laoshan Laboratory, Qingdao, 266237 China

**Keywords:** Ecology, Ocean sciences

## Abstract

Assessing the health of coastal ecosystems is crucial for maintaining ecological balance. One significant indicator of water eutrophication is the distribution of chlorophyll-*a* (Chl-*a*), which makes obtaining a complete three-dimensional spatial distribution of Chl-*a* essential for assessment. This study utilized the linear radial basis function (RBF-Linear) method to obtain a comprehensive and reasonable spatial distribution of Chl-*a*. The method was applied to obtain the three-dimensional spatial field of Chl-*a* concentration in the Bohai Sea in March, May, August, and October from 2016 to 2018. The distribution pattern of Chl-*a* concentration in the Bohai Sea displayed characteristic spatial and temporal variations. Spatially, high Chl-*a* concentration was most concentrated in coastal waters, particularly in estuaries and mariculture areas. Temporally, there were two peaks in March and August. The total Chl-*a* and areas with high Chl-*a* concentration in four sub-regions of the Bohai Sea were also calculated to enable a comprehensive assessment of the marine ecological environment. By analyzing the temporal and spatial variation of Chl-*a* in the Bohai Sea and evaluating the marine ecological environment, we confirmed the feasibility and rationality of RBF-Linear. Our findings have the potential to contribute to improve the accuracy of ecological models and assessment of the satellite products.

## Introduction

Phytoplankton, as a primary producer, play a crucial role in maintaining marine ecosystems^1^. However, anthropogenic activities have resulted in ecological disasters such as red tide and green tide caused by imbalanced phytoplankton blooms^2^. Currently, phytoplankton monitoring is largely dependent on the estimation of chlorophyll-*a* (Chl-*a*, μg/L) concentration, which is quantified by satellite data and field sampling^3,4^. Despite their efficiency, both approaches have limitations that affect their accuracy and reliability. Satellite data suffer from inadequate space coverage due to cloud occlusion or orbit gaps, and the uncertainty of satellite products in muddy waters is relatively large^5–7^. Field sampling, while yielding more accurate data, is spatially discontinuous, making it challenging to conduct comprehensive spatial–temporal studies of Chl-*a*. To overcome these shortcomings, spatial interpolation techniques are employed to complement the information obtained from field observations, thereby improving the reliability of the conclusions derived from such data.

In numerous scientific disciplines, interpolation and fitting of dispersed data are fundamental duties. Radial basis function (RBF)^8–10^, Kriging Interpolation ^11–13^ and inverse distance weighting (IDW) ^13,14^ and other interpolation methods are widely used in various fields such as fluid mechanics, geology and meteorology. These techniques demonstrate excellent performance in two-dimensional spatial interpolation, but their application in three-dimensional spatial interpolation is still limited, necessitating additional research to develop trustworthy and effective three-dimensional spatial interpolation techniques.

The Bohai Sea, the largest semi-enclosed sea in China, possesses an average depth of ~ 18 m ^7^. However, it is recognized as one of the world's muddiest oceans, which lead to significant overestimation of standard satellite Chl-*a* products ^15^. Nevertheless, satellite-based Chl-*a* measurements are unable to capture the comprehensive three-dimensional Chl-*a* spatial fields since they solely collect surface layers. A highly competent spatial interpolation method is crucial to estimate the entire Chl-*a* three-dimensional space field. Based on the observations of the Bohai Sea in August 2017, the best interpolation approach was determined via the linear radial basis function alongside the ten-fold cross-validation, correlation analysis, and spatial distribution results ^16^. This study uses rare high-density in-situ Chl-*a* measurements in the Bohai Sea, which were collected between 2016 and 2018, to interpolate vast amounts of observation data. The reconstructed Chl-*a* images provide insights into the temporal and spatial variations, thereby assessing the marine ecological environment of four Bohai Sea sub-regions, namely Bohai Bay (BHB), Liaodong Bay (LDB), Laizhou Bay (LZB), and central Bohai Sea (CBS). Moreover, this interpolation technique has the potential to evaluate satellite products and enhance ecological model accuracy, as supported by additional evidence.

## Data and methods

### Observation data

**Figure 1:**
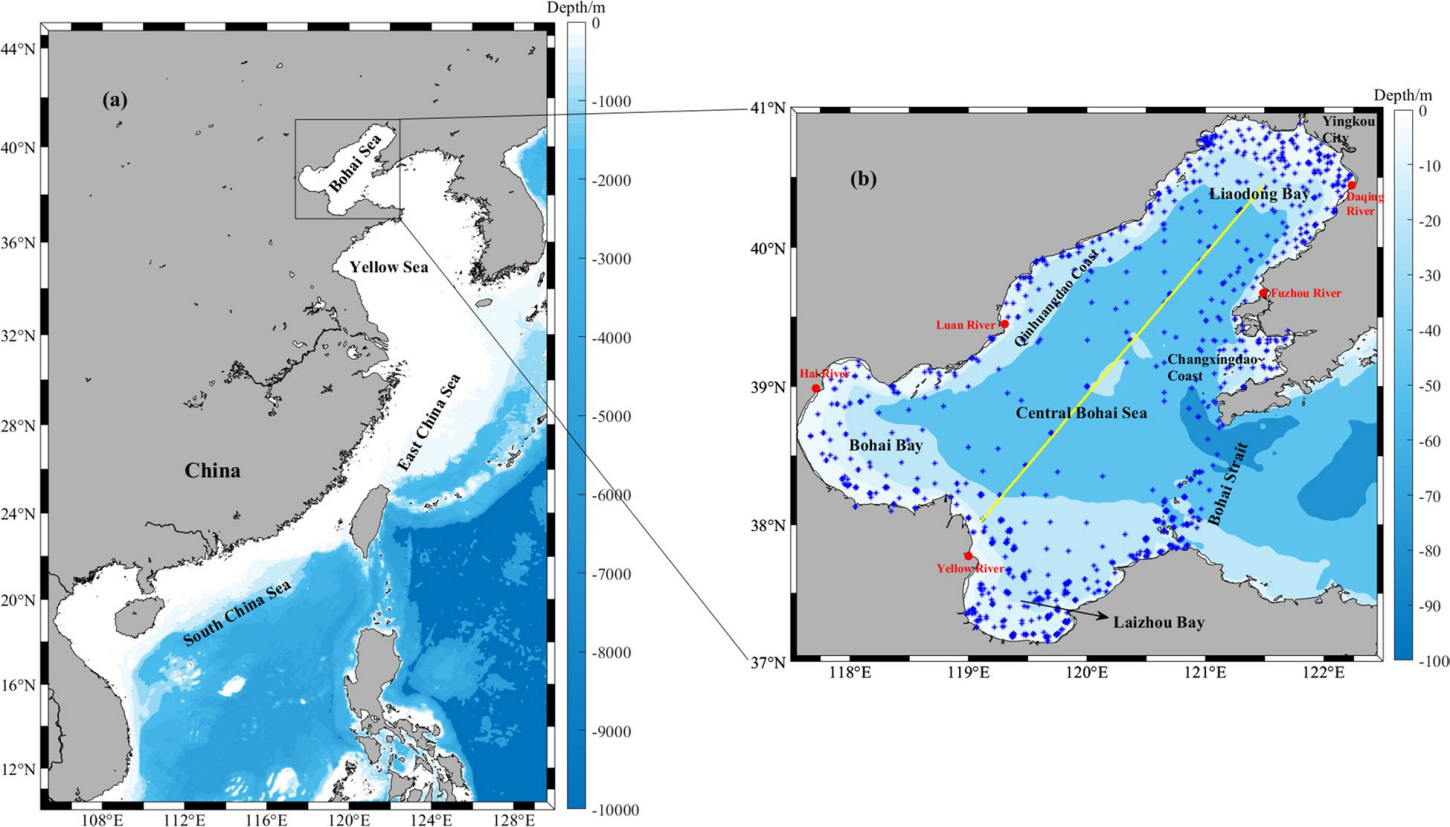
Location (**a**) and bathymetry (**b**) of the Bohai Sea. The observation locations are represented by blue stars. The solid yellow line is the section position selected in the subsequent vertical distribution diagram. This figure is drawn using the m-map toolbox in Matlab R2019b.

This paper utilizes data collected from in-situ observations of Chl-*a* concentration in the Bohai Sea during four distinct seasons—early spring, late spring, summer, and autumn—spanning from 2016 to 2018. The data was sourced from the *National Marine Environment Monitoring*. This study's sample method focused mainly on increasing the number of sampling points along the coast, while decreasing the number of sampling points in the central Bohai Sea due to the difficult sampling conditions there (as depicted in Fig. 1). Surface (< 2 m), middle (~ 10 m), and bottom (> 15 m) layers of observational data were used. The number of observation points at different layers in each characteristic month from 2016 to 2018 is shown in Table 1.

**Table 1:** The number of sampling points in surface, middle and bottom layers of Bohai Sea during March, May, August and October, 2016–2018.

	Surface layer			Middle layer			Bottom layer		
	2016	2017	2018	2016	2017	2018	2016	2017	2018
March	290	271	384	85	77	82	7	1	1
May	370	436	489	86	131	139	13	20	19
August	368	432	518	99	134	135	13	22	22
October	365	429	460	99	157	115	14	17	10

The Chl-*a* concentration is measured as follows: water samples are filtered using a Whatman GF/F glass microfiber filter under vacuum conditions of less than 5 × 10^4^ Pa. The water sample volume is 200 mL in the medium nutrient area, and 300 mL to 500 mL in the relatively clear area. The filters are immediately analyzed with a laboratory fluorometer (Turner Designs, TD-700), otherwise stored in a refrigerator (−20℃) for laboratory analysis^17^. The fundamental idea behind the fluorescence method is that when blue light is used to stimulate the chloraacetone extract, red light is produced. The phytoplankton is obtained by extracting and filtering a specific volume of saltwater using 90% acetone, and a fluorescence meter is then used to measure the extraction solution's fluorescence before and after acidification. The experiment's implementation standard complies with *the Code of Practice for Marine Monitoring Technology. Part 5: Marine ecology of China *(*HY/T 147.5-2013*).

### Three-dimensional interpolation method

#### Radial basis function fitting

The technique of scatter interpolation commonly resorts to the utilization of the Radial Basis Function (RBF) fitting approach. This method has been observed to possess a notable benefit of being unrestricted by dimensionality, as well as being capable of providing minimum variance estimates that are statistically unbiased^8,18,19^.

The observed data set is recorded as *D*(*x*_*i*_*, y*_*i*_*, z*_*i*_)*, i* = *1, 2, …, n*, where (*x*_*i*_*, y*_*i*_) is obtained from the longitude and latitude of point *i* transformed into a plane rectangular coordinate system (using Miller map projection). *z*_*i*_ is the depth, and *n* is the number of observation points. Because Chl-*a* concentration typically spans large orders of magnitude, logarithmic transformation is performed on Chl-*a* data before fitting, i.e. *D'* = *log10*(*D*)^15,20,21^. It should be noted that the interpolated *log10(D)* value is converted back to the *D* value before the interpolation error is calculated. Assuming isotropic distribution of Chl-*a* concentration, the formula for calculating points $\widetilde{D}$(*x*_*j*_*, y*_*j*_*, z*_*j*_) to be interpolated is as follows:

1 $$\widetilde{D}({x}_{j},{y}_{j},{z}_{j})=\sum_{i=1}^{n}{\beta }_{i}\varphi ({d}_{ij})+{\lambda }_{1}+{\lambda }_{2}{x}_{i}+{\lambda }_{3}{y}_{i}+{\lambda }_{4}{z}_{i}$$

The *β*_*i*_, *λ*_*1*_, *λ*_*2*_, *λ*_*3*_ and *λ*_*4*_ are calculated as follows:

2 $$\left[\begin{array}{l}\beta \\ \lambda \end{array}\right]={\left[\begin{array}{cc}A& P\\ {P}^{T}& 0\end{array}\right]}^{-1}\cdot \left[\begin{array}{c}{D}^{^{\prime}}\\ 0\end{array}\right]$$

where,

3 $$\beta =\left[\begin{array}{l}\begin{array}{l}{\beta }_{1}\\ {\beta }_{2}\\ \vdots \end{array}\\ {\beta }_{n}\end{array}\right], \lambda =\left[\begin{array}{l}\begin{array}{l}{\lambda }_{1}\\ {\lambda }_{2}\\ {\lambda }_{3}\end{array}\\ {\lambda }_{4}\end{array}\right], P=\left[\begin{array}{cc}\begin{array}{l}1\\ \begin{array}{l}1\\ \begin{array}{l}\vdots \\ 1\end{array}\end{array}\end{array}& \begin{array}{l}{x}_{1}\\ {x}_{2}\\ \begin{array}{l}\vdots \\ {x}_{n}\end{array}\end{array}\end{array} \begin{array}{cc}\begin{array}{l}{y}_{1}\\ {y}_{2}\\ \begin{array}{l}\vdots \\ {y}_{n}\end{array}\end{array}& \begin{array}{l}{z}_{1}\\ {z}_{2}\\ \begin{array}{l}\vdots \\ {z}_{n}\end{array}\end{array}\end{array}\right], {D}^{^{\prime}}=\left[\begin{array}{l}\begin{array}{l}{D}^{^{\prime}}({x}_{1},{y}_{1},{z}_{1})\\ {D}^{^{\prime}}({x}_{2},{y}_{2},{z}_{2})\\ \vdots \end{array}\\ {D}^{^{\prime}}({x}_{n},{y}_{n},{z}_{n})\end{array}\right]$$

4 $$A=\left[\begin{array}{ccc}\begin{array}{l}\begin{array}{cc}0& \varphi ({d}_{12})\end{array}\\ \begin{array}{cc}\varphi ({d}_{21})& 0\end{array}\end{array}& \begin{array}{l}\varphi ({d}_{13})\\ \varphi ({d}_{23})\end{array}& \begin{array}{l}\begin{array}{cc}\dots & \varphi ({d}_{1n})\end{array}\\ \begin{array}{cc}\dots & \varphi ({d}_{2n})\end{array}\end{array}\\ \vdots & \ddots & \vdots \\ \begin{array}{cc}\varphi ({d}_{n1})& \varphi ({d}_{n2})\end{array}& \cdots & 0\end{array}\right]$$

where *d*_*ij*_ is the Euclidean distance between (*x*_*i*_*, y*_*i*_*, z*_*i*_) and (*x*_*j*_*, y*_*j*_*, z*_*j*_). *φ*(*d*_*ij*_) is the basis function. There are many calculation methods of RBFs. The following three RBFs are contemplated herein: (1) Linear: $\varphi ({d}_{ij})={d}_{ij}$; (2) Gaussian: $\varphi ({d}_{ij})={e}^{-0.5\cdot {d}_{ij}^{2}/{\varepsilon }^{2}}$; (3) Multi-quadrics: $\varphi ({d}_{ij})=\sqrt{1+{d}_{ij}^{2}/{\varepsilon }^{2}}$. In this study, there exists a constant *ε* in the basis function, which is inversely proportional to the critical radius and is set to 0.1^9^.

As shown in Table 2, Chl-*a* concentration is anisotropically distributed, meaning that vertical variation is larger than horizontal variation; the vertical Chl-*a* concentration change rate is 2–4 times greater than the horizontal change rate. As Miller projection is adopted in the horizontal direction, the horizontal distance between the two points is reduced to ~ 10^–6^ before projection and the change rate is raised by about 10^6^ times. Therefore, the rate of change in the horizontal direction is 2–4 orders of magnitude higher than that in the vertical direction in the calculation process. Through some tests, we increased the weight of the change rate in the vertical direction (*z*_*i*_*’* = *z*_*i*_/1000) in the calculation process, aiming to keep the change rate of all dimensions as consistent as possible to obtain better interpolation results.

**Table 2:** The range of absolute rate of change of Chl-*a* concentration in horizontal and vertical directions.

Directions	The range of absolute rate of change (μg L^−1^ m^−1^)
Horizontal direction	~ 10^–9^ to 10^0^
Vertical direction	~ 10^–5^ to 10^2^

#### Inverse distance weight interpolation

The inverse distance weighted (IDW) interpolation method determines the weight according to the distance between the observation data *D*(*x*_*i*_*, y*_*i*_*, z*_*i*_) and the interpolation points $\widetilde{D}$(*x*_*j*_*, y*_*j*_*, z*_*j*_), and calculates the values of the points to be interpolated^14^. (*x*_*i*_*, y*_*i*_) is obtained from the longitude and latitude of point *i* transformed to the plane cartesian coordinate system (using Miller map projection). *z*_*i*_ is the depth and *n* is the number of observation points. The formula for calculating the Chl-*a* concentration value $\widetilde{D}$(*x*_*j*_*, y*_*j*_*, z*_*j*_) of the estimated point is as follows:

5 $$\widetilde{D}({x}_{j},{y}_{j},{z}_{j})=\sum_{i=1}^{n}\frac{D({x}_{i},{y}_{i},{z}_{i})}{{{d}_{ij}}^{\alpha }}/\sum_{i=1}^{n}\frac{1}{{{d}_{ij}}^{\alpha }}$$

where, ${d}_{ij}=\sqrt{{({x}_{j}-{x}_{i})}^{2}+{({y}_{j}-{y}_{i})}^{2}+{({z}_{j}-{z}_{i})}^{2}}$. *α* is set to 3.

#### Kriging interpolation

Kriging interpolation began in the mining industry in the early 1950s as a method for estimating ore reserves and has since become widely used as a spatial interpolation method^22^. Kriging is founded on a concept of random functions with the surface or volume assumed one realization of a random function with a known spatial covariance. Regionalized variable theory assumes that the spatial variation of any variable can be expressed as the sum of the following three components: (a) A structural component having a constant mean or trend. (b) A regionalized variable, which is the random but spatially correlated component. (c) A random but spatially uncorrelated noise or residual component. In this study, Kriging interpolation is implemented using MATLAB toolbox^23^.

### Evaluation indicators

In this paper, the best interpolation method is determined by cross validation of ten folds^24^, and the testing process is as follows: according to the random order of data, 1, 2, …, 10 are taken as the starting point respectively, and one is taken from every 10 points to form the dataset to be interpolated. The remaining points serve as interpolation reference points and then ten experiments are carried out for each interpolation method. The Mean Absolute Error (MAE) and Mean Relative Error (MRE) of each interpolation method are calculated by comparing the estimated value with the original value. MAE and MRE are calculated as follows:

6 $$MAE=\sum_{i=1}^{n}\left|{D}_{i}-\widetilde{{D}_{i}}\right|/n$$

7 $$MRE=\sum_{i=1}^{n}\frac{\left|{D}_{i}-\widetilde{{D}_{i}}\right|}{{D}_{i}}/n$$

In addition, Pearson correlation coefficient *r* is used to further determine the correlation between interpolation results and original data. The calculation formula is as follows:

8 $$r=\frac{1}{n}\sum_{i=1}^{n}\{[{log}_{10}({D}_{i}-\overline{{D }_{i}})/{{{log}_{10}(D}_{i}}_{SD})]\times [{log}_{10}(\widetilde{{D}_{i}}-\overline{\widetilde{{D }_{i}}})/{log}_{10}({\widetilde{{D}_{i}}}_{SD})]\}$$

In the formula (6–8), ${D}_{i}$ is the observed value, $\widetilde{{D}_{i}}$ is the estimation of interpolation, and *n* is the number of interpolation points. $\overline{{D }_{i}}$ and $\overline{\widetilde{{D }_{i}}}$ are the mean value of ${D}_{i}$ and $\widetilde{{D}_{i}}$. ${{D}_{i}}_{SD}$ and ${\widetilde{{D}_{i}}}_{SD}$ are the standard deviation of ${D}_{i}$ and $\widetilde{{D}_{i}}$ respectively.

## Results

**Figure 2:**
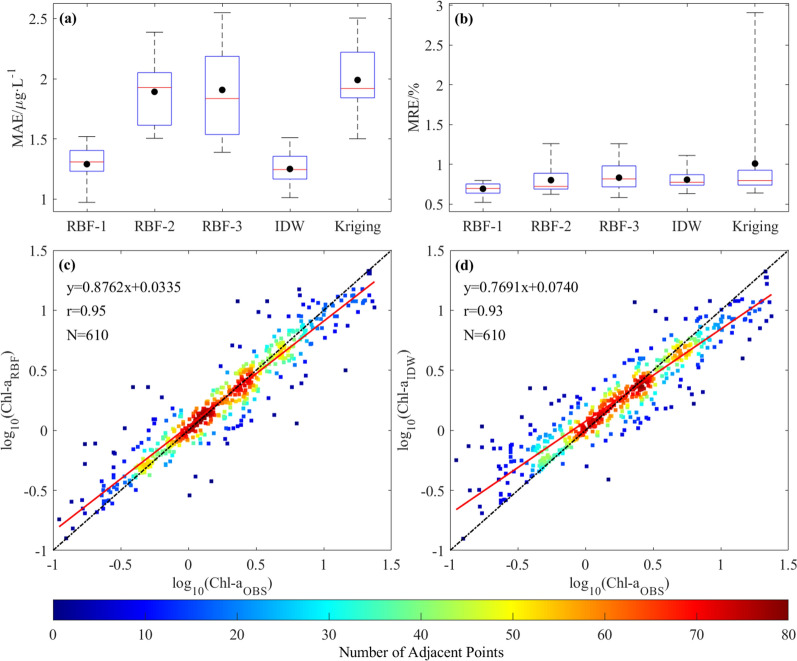
MAE and MRE results of ten-fold cross-validation (**a, b**). RBF-1, RBF-2 and RBF-3 are RBF-Linear, RBF-Gaussian and RBF-Multi quadrics, respectively. On each box, the red center mark represents the median, and the bottom and top edges of the box represent the 25th and 75th percentiles, respectively. The two extreme data points to which the whiskers extend are the minimum and the maximum. The black dots represent the average of the ten experimental groups. The scattering diagram of RBF-Linear and IDW results after logarithm taking, respectively (**c, d**). The coded colors represent relative densities. The solid red line is the regression line. The black dotted line is y = x.

Figure 2a, b shows all verification results of the ten-fold cross-validation. Notably, the obtained results illustrate a considerable decrease in mean absolute error (MAE) of RBF-Linear and IDW interpolation methods compared to the other three approaches. Specifically, the reduction in MAE for these methods is approximately 33%. Additionally, RBF-Linear interpolation method demonstrates a notable decrease of around 32% in mean relative error (MRE) compared to other methods. Furthermore, the interspersed evaluation indices of the RBF-Linear and IDW interpolation methods in ten groups of interpolation results depict stability with minimal fluctuation, indicating these methods are relatively robust to the variability of Chl-*a* concentration change rates, observation point distribution, and other pertinent factors. To conclude, the RBF-Linear and IDW methods illustrate superior performance compared to the other three methods. The subsequent comparison and evaluation of both methods will be conducted through correlation analysis and spatial distribution analysis.

The correlation between interpolation results and observed values is shown in Fig. 2c, d. The findings of this study establish a noteworthy and substantial association between the interpolation results of the two methods and the observed values, with a correlation coefficient of 0.95 and 0.93 (p < 0.01), respectively. Additionally, the slope of the regression line between the RBF-Linear interpolation outcome and the observed value exhibits a greater similarity to 1 when compared to that of the IDW interpolation outcome. In summary, based on the analysis of evaluation indexes, RBF-Linear interpolation method is slightly superior to IDW, but has no obvious advantages.

**Figure 3:**
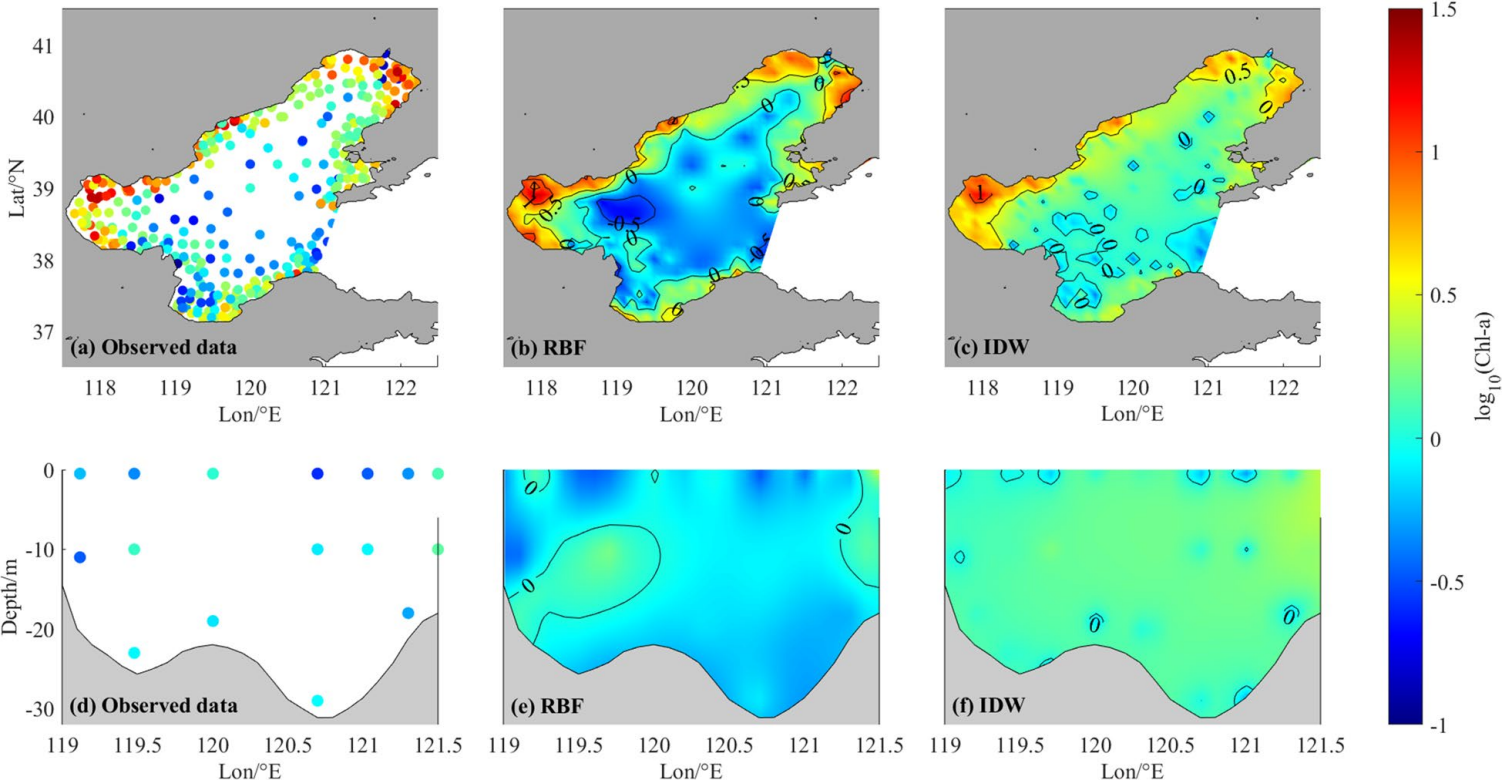
The first row is the horizontal distribution of log_10_(Chl-*a*) in the surface layer, which is the observation data (**a**), RBF results (**b**) and IDW results (**c**) respectively. The second row is the vertical distribution of log_10_(Chl-*a*), which is the observation data (**d**), RBF results (**e**) and IDW results (**f**) respectively. The location of selected section is shown in Fig. 1.

The three-dimensional spatial interpolation method requires a comprehensive dataset that encompasses all three-dimensional spatial field data while ensuring the most reasonable spatial distribution. In this study, we conducted further research and analysis of the spatial distribution of RBF-Linear and IDW interpolation results (Fig. 3). Our findings reveal that both RBF-Linear and IDW interpolation results accurately depict the horizontal and vertical distribution of Chl-*a* concentration. However, IDW exhibits less smoothness compared to RBF-Linear and a noticeable bull-eye pattern in both horizontal and vertical distribution. Based on our analysis, we concluded that RBF-Linear acts as the optimal method for reconstructing the three-dimensional spatial field of in-situ measured Chl-*a* concentration in the Bohai Sea.

## Discussion

As shown in Table 1, the number of observation points in this paper exceeds 300 every month, far more than that in previous studies^25–29^. Based on the comparisons using substantive dataset, we have selected RBF-Linear as the most effective interpolation method. In this paper, we seek to scrutinize the appropriateness of this approach within practical applications.

### Spatial and temporal distribution of Chl-*a* concentration in the Bohai Sea

**Figure 4:**
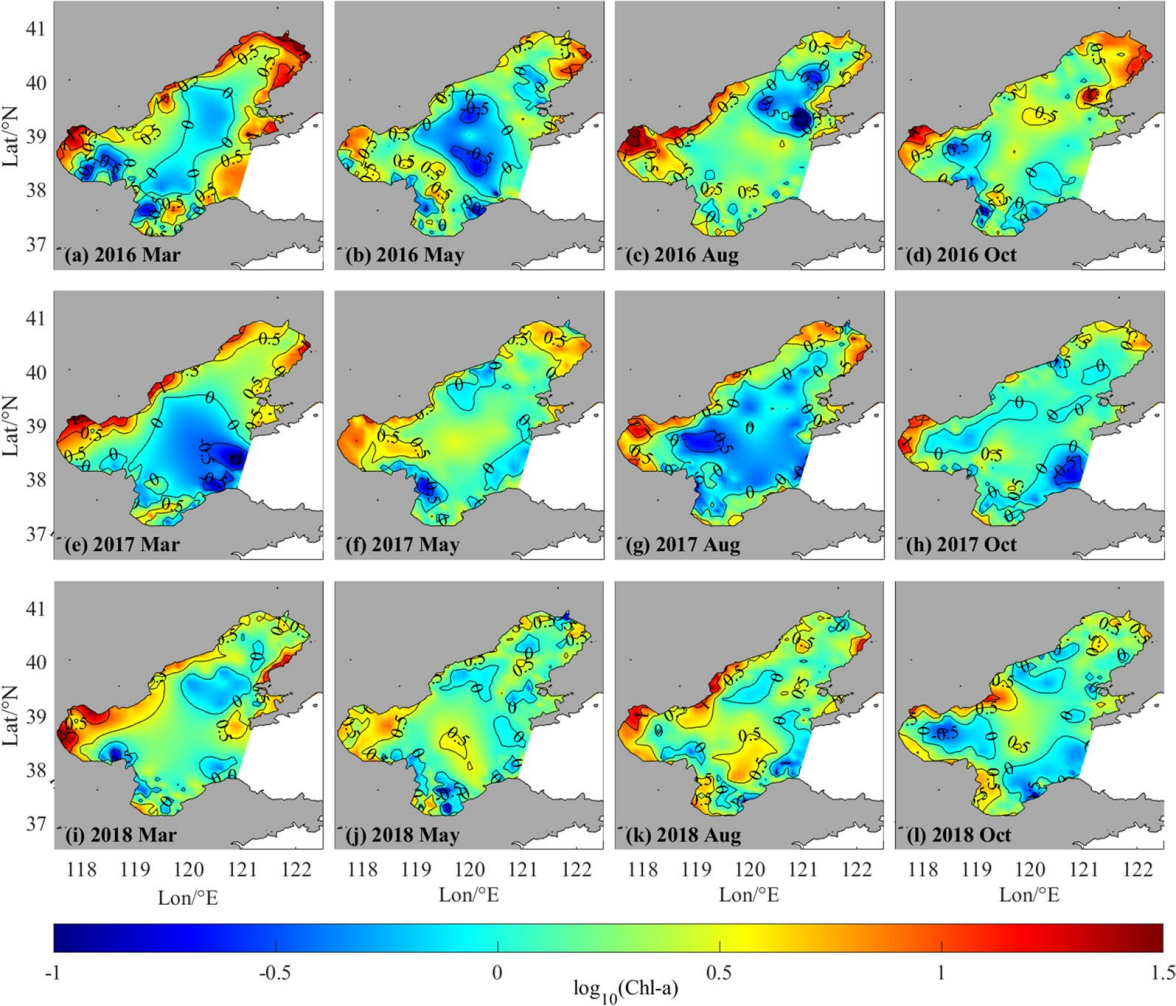
Spatio-temporal distribution of log_10_(Chl-*a*) in surface layer in Bohai Sea of March, May, August and October from 2016 to 2018.

**Figure 5:**
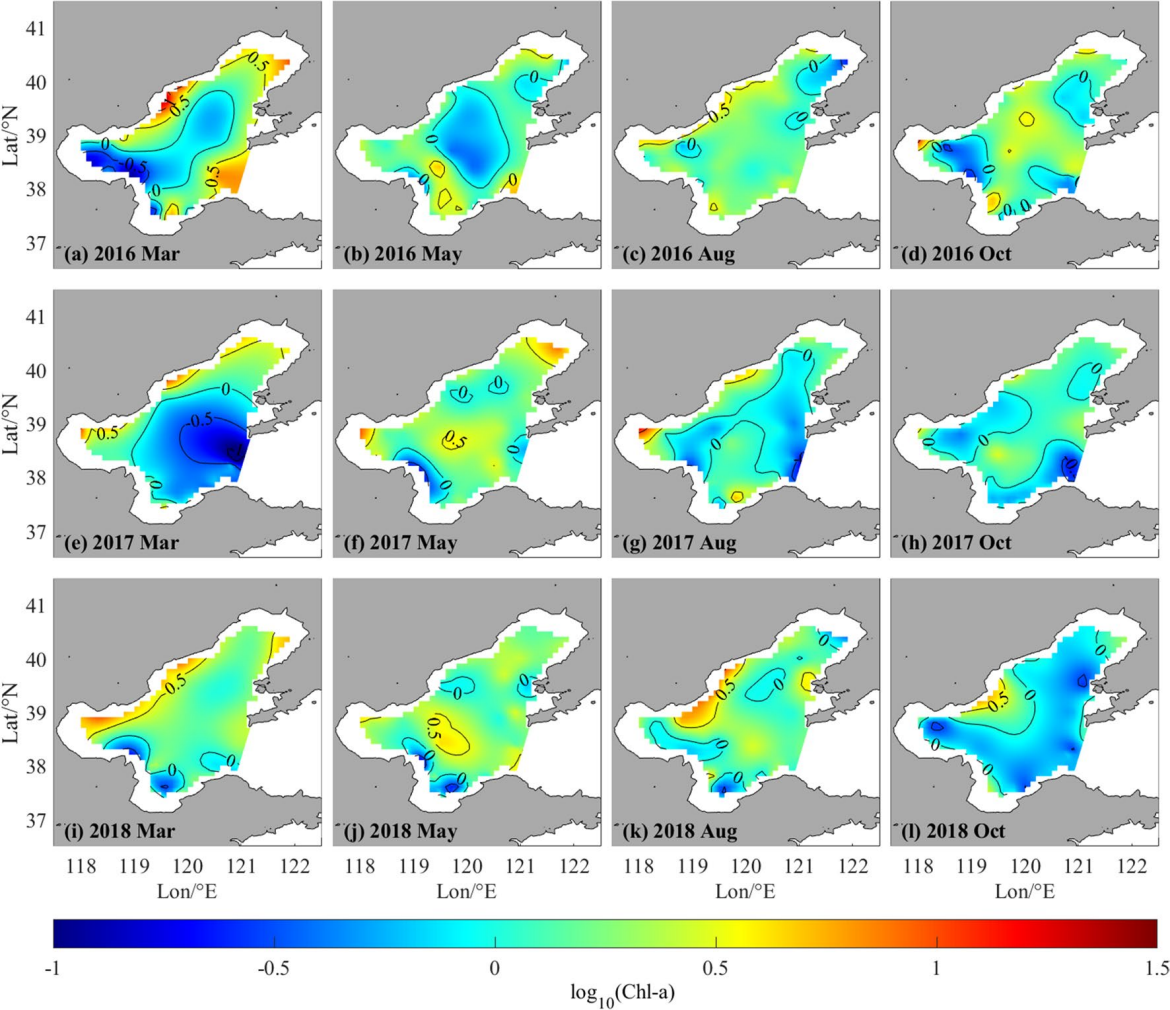
Spatio-temporal distribution of log_10_(Chl-*a*) in 10 m layer in Bohai Sea of March, May, August and October from 2016 to 2018.

The spatial distribution of Chl-*a* concentration in the Bohai Sea during March, May, August, and October of 2016 to 2018 was investigated using RBF-Linear interpolation (Figs. 4, 5). The results show that Chl-*a* concentration in the coastal area was generally higher than that in the offshore area, and the concentration in the surface layer (0.5 m) was higher than that in the middle layer (~ 10 m). Chl-*a* concentrations over 10 μg/L were found primarily in surface water of BHB and LDB, particularly in estuaries such as the Haihe River, Luanhe River, Fuzhou River, and Daqing River, and in regions used for mariculture, e.g., Qinhuangdao coastal waters. This pattern is attributed to the influence of nutrient inputs from coastal rivers and maricultural activities that encourage the growth of phytoplankton in coastal seas^30–34^. Transparency of the water also plays a crucial role in determining the depth of the euphotic layer, which in turn limits the depth at which phytoplankton can grow^25,35^. Thus, the primary growth of phytoplankton takes place in surface and subsurface water. In some sections of the Bohai Sea's 10 m layer, scattered throughout the BHB, LDB, and Qinhuangdao offshore water, Chl-*a* concentration is near to 10 μg/L.

The temporal distribution of Chl-*a* in the Bohai Sea exhibits seasonal variation, as evident from Figs. 4 and 5. The Chl-*a* concentration is notably higher in March and August relative to the other months, owing to the availability of key factors that promote phytoplankton growth. Specifically, sufficient nutrients and appropriate levels of photosynthetically active radiation (PAR) during March promote the growth and development of phytoplankton, thereby contributing to the elevated levels of Chl-*a* observed during this period. The Chl-*a* concentration in August is influenced by the availability of nutrients, particularly rainfall, which stimulates phytoplankton bloom^25,36^. The inter-annual variability in the Chl-*a* concentration of the Bohai Sea is also evident during the same period. The Bulletin on the State of China's Marine Ecology and Environment reports a significant decline in the concentration of Chl-*a* during March and August of 2017 and 2018 as compared to the corresponding months in 2016. This decline may be attributed to a comparative decrease in the input of nutrients during 2017 and 2018.

In summary, the investigation of the spatial and temporal pattern of Chl-*a* concentration in the Bohai Sea reveals a substantial improvement in the marine ecological condition during the years 2017–2018. Nevertheless, areas proximal to aquaculture farms and estuaries have not witnessed similar progress. The findings demonstrate the correspondence between the interpolated Chl-*a* distribution and a range of actual physical and biochemical variables, hence confirming the validity of the RBF-Linear interpolation technique for reconstructing the spatial and temporal Chl-*a* distribution.

### Marine ecological environment assessment in four sub-regions of Bohai Sea

Total amounts of Chl-*a* and high Chl-*a* area are two crucial indicators for evaluating the marine ecosystem. Comparing the total amounts of Chl-*a* and the high Chl-*a* area in four Bohai Sea subregions (BHB, LDB, LZB, and CBS), this section examines whether it is reasonable to calculate these two indicators using RBF-Linear interpolation results.

**Figure 6:**
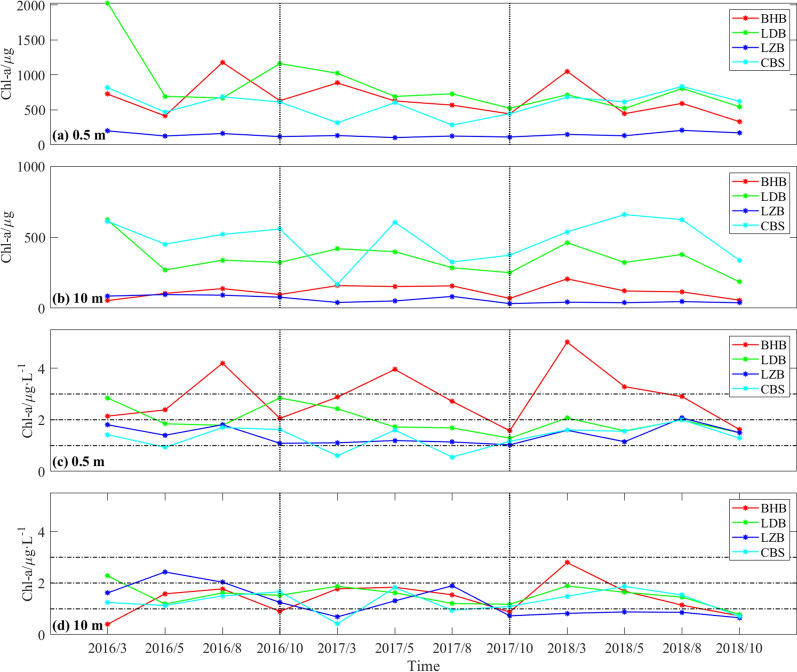
The changes of total Chl-*a* in the surface layer and the 10 m layer based on the integration of observed mean values over time, respectively (**a, b**). And the changes of total Chl-*a* of the surface layer and 10 m layer over time based on the integration of interpolation results, respectively (**c, d**).

Figure 6 illustrates the temporal variations of total amounts of Chl-*a* in four subregions of the Bohai Sea. The investigation employs two distinct methods for computing the total amounts of Chl-*a*. The first approach involves the multiplication of the average Chl-*a* concentration obtained from observations by each subregion's area, while the second method involves the integration of the interpolated Chl-*a* results. The findings (Fig. 6a, b) reveal that the first method yields remarkably higher values than the second method. The overestimation of total amounts of Chl-*a* using the first method can be attributed to the extensive range of Chl-*a* concentrations in the dataset, with maximum and minimum values varying by more than two orders of magnitude. In contrast, the interpolation method offers a more accurate approach to mitigate this issue.

The study examines the temporal fluctuation of total amounts of Chl-*a* in the surface of four sub-regions. The results are presented in Fig. 6c, which illustrates significant variations in total amounts of Chl-*a* among the sub-regions from 2016 to 2018. Specifically, the total amounts of Chl-*a* in BHB exhibits a yearly increase in March from 2016 to 2018, while LDB experiences a yearly decrease. Meanwhile, LZB and CBS display slight fluctuations. In May, the total amounts of Chl-*a* show little variation across all sub-regions. In August, LDB shows minimal change throughout the three-year period, while the total amounts of Chl-*a* in BHB declines substantially in 2017 and 2018, compared to 2016. Further, it is observed that the typhoon "Wimbiya" in August 2018 results in a significant increase in Chl-*a* concentrations in LZB and CBS, with subsequent rise of dissolved inorganic nitrogen and silicon in the surface^29^. Finally, the total amounts of Chl-*a* in BHB and LDB dramatically decreases in October over the three-year period, with no significant changes noted in LZB and CBS.

Figure 6d depicts total amounts of Chl-*a* at 10 m layer in the four sub-areas, which is noticeably less than that at the surface. The findings demonstrate that BHB experienced an upward trend in total amounts of Chl-*a* in March, while LZB witnessed a decline over time. In contrast, LDB and CBS exhibited no significant changes in total amounts of Chl-*a*. Notably, the total amounts of Chl-*a* in CBS increased dramatically in 2018, while the other three sub-regions remained relatively stable. In August, the total amounts of Chl-*a* in three sub-regions remained relatively constant except for a notable fluctuation in CBS. However, in October, the total amounts of Chl-*a* showed a yearly decline across all four sub-regions.

In light of the total amounts of Chl-*a* concentrations, it is evident that the marine ecological environment underwent significant changes among different regions and seasons during the years 2016 to 2018. During the onset of spring, the marine ecological environment of BHB deteriorated, while that of LDB improved considerably. Additionally, the marine ecological environment of CBS was at risk of deterioration towards late spring. During the summer, the marine ecological environment of BHB witnessed substantial improvement, while in certain areas, remarkable ecological improvements were observed in autumn. These shifts in primary production in the three bays and CBS are primarily attributed to variations in water clarity and nutrient composition, as documented in previous research^26^. The findings also emphasize the significant impact of typhoons on the marine ecology. It is worth noting that marine ecological studies, primarily focusing on the surface and subsurface layers, tend to ignore the concentration of Chl-*a* in deep layers. However, our research reveals notable differences in Chl-*a* concentrations in the deep layer among areas, which might relate to water depth. Therefore, while disregarding the Chl-*a* concentration in the deep layer may be possible in several places during assessing the marine ecological environment, it is necessary to consider it in particular areas such as CBS.

The area where algal blooms occur is a crucial indicator of the quality of the marine biological environment in a region. Chl-*a* concentrations that exceed the threshold of 10 μg/L are widely considered a critical limit for phytoplankton blooms in the marginal seas of China^37^. This investigation focuses on scrutinizing and assessing the marine ecological environment in four sub-regions of the Bohai Sea by evaluating the extent of areas with high concentrations of Chl-*a* (> 10 μg/L).

**Figure 7:**
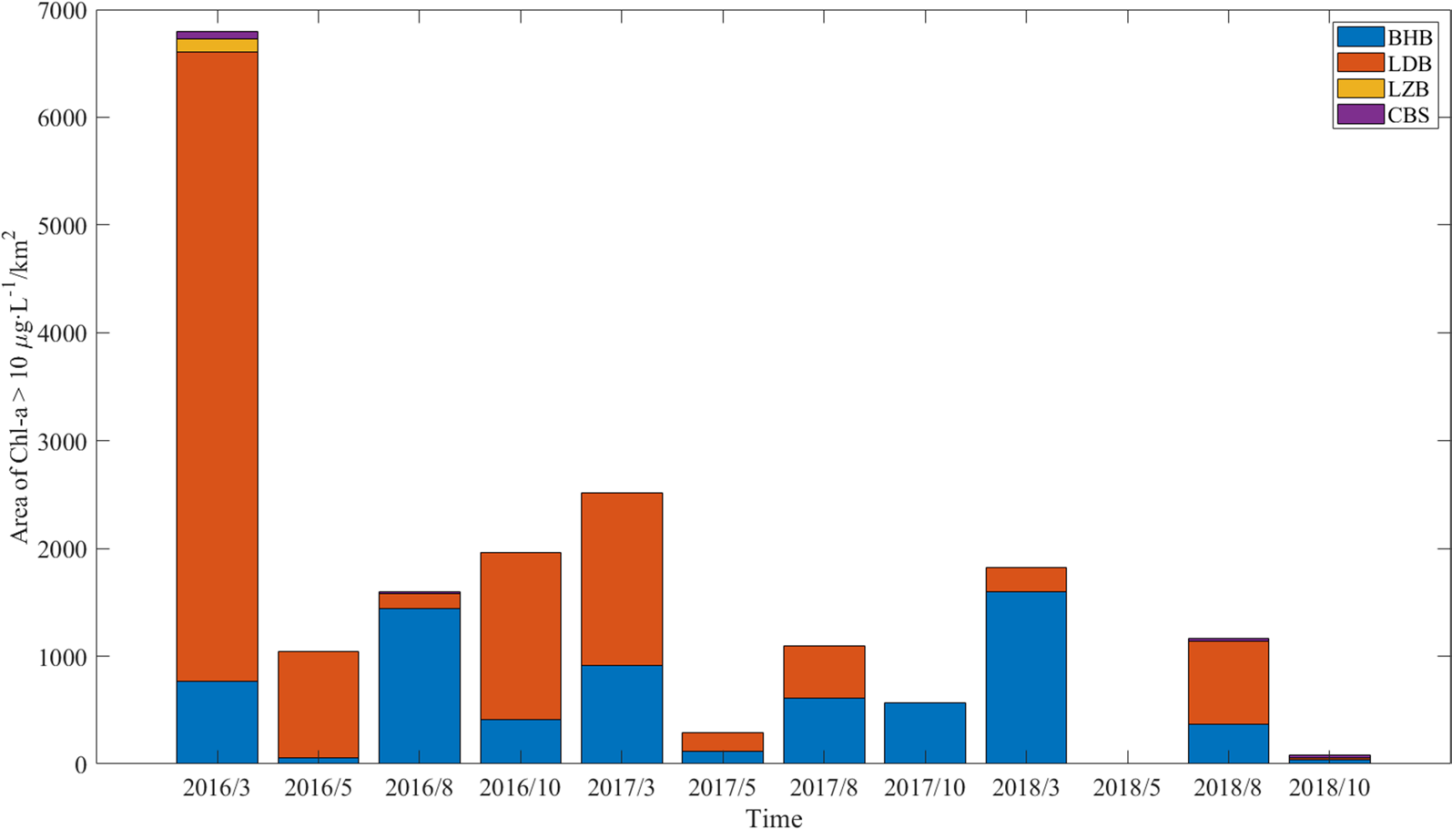
The area of high Chl-*a* concentration in the surface layer (Chl-*a* > 10 μg/L). The height of each bar is the sum of its elements.

In Fig. 7, the high Chl-*a* concentration region and its four sub-regions in the Bohai Sea are presented. The results revealed that the area with high Chl-*a* concentration has decreased annually from 2016 to 2018. It is worth noting that the area calculated in this study demonstrates the extent of the region reaching the critical value of the bloom rather than the area of the disastrous bloom. Consequently, the area estimated in this study was more extensive than the data provided in the *China Marine Disaster Bulletin* (Table 3). Nonetheless, the changes observed in the high Chl-*a* concentration area estimated by interpolation results were consistent with the variation in data given in the *China Marine Disaster Bulletin*. These results confirm the validity of RBF-Linear interpolation findings and the use of the high Chl-*a* concentration area to evaluate the marine ecological environment in the Bohai Sea. Moreover, the *Bulletin of the State of the Marine Ecological Environment of China* reported a consistent yearly decrease in total nitrogen and phosphorus discharged into the Bohai Sea from 2016 to 2018, which aligned with the variation in high Chl-*a* concentration region. These findings indicate that the reduction in nutrient input played a significant role in ameliorating the water quality of the Bohai Sea.

**Table 3:** The area of red tide, total nitrogen and total phosphorus in Bohai Sea from 2016 to 2018.

Time	The area of red tide (km^2^)	Total nitrogen (tons)	Total phosphorus (tons)
2016	740	5371	317
2017	342	4327	190
2018	62	3717	59

Figure 7 also displays the changes in Chl-*a* concentrations exceeding 10 μg/L on the surface of the four sub-regions of the Bohai Sea from March, May, August, and October in 2016 to 2018. The surface waters of LDB and BHB exhibited prevalent Chl-*a* concentrations exceeding 10 μg/L. BHB is surrounded by various megacities, including Tianjin, and is fed by 16 rivers. Water exchange in the region can be impacted by reclamation and mariculture activities, along with the combined influence of land runoff and offshore reclamation. As a result, BHB's coastal water quality is declining, and red tides are frequent ^27^. On the other hand, LDB, which is encircled by numerous industrial cities, is mainly affected by land-based pollution, leading to significant phytoplankton proliferation ^36^. The surface waters of BHB exhibited a high Chl-*a* content over a large area (> 800 km^2^) in March and August from 2016 to 2018, with no noticeable interannual fluctuations. Conversely, there was a significant decrease in LDB, stemming primarily from the slow growth of heavy industry in Hebei and Liaoning Province and the significant reduction of land-based pollution, leading to a reduction in Chl-*a* concentration ^36^. The high Chl-*a* content area on the surface waters of LZB and CBS is frequently small. LZB is situated in the southern Bohai Sea, bordered by the Yellow River, the Xiaoqing River, the Weifang River, and the Jiaolai River, and the ecological environment in the region is primarily influenced by river runoff, domestic sewage, and mariculture. Consequently, both the Chl-*a* concentration and the area with high Chl-*a* concentration are generally lower than those in BHB and LDB ^38^. Furthermore, because the dissolved inorganic nitrogen in CBS is relatively low compared to other regions ^39^, the Chl-*a* concentration and the area with high Chl-*a* concentration in this region are relatively low ^40^. The investigation reveals that the extent of the high Chl-*a* concentration area, exceeding 10 μg/L, in the 10 m layer is primarily localized in the BHB and LDB sub-regions of the Bohai Sea, encompassing an area which is typically less than or equal to 100 km^2^, despite a noteworthy anomaly in March 2016 where the high Chl-*a* concentration zone in LDB is calculated to be approximately 300 km^2^. Owing to the relatively diminutive magnitude of high Chl-*a* concentration areas encountered in the 10 m layer across each sub-region of the Bohai Sea, we refrain from comprehensive analysis in the present study.

Based on the investigation conducted into the marine ecological environment of the Bohai Sea and its four sub-regions, our findings reveal that the BHB sub-region shows no significant improvement, whereas the LDB sub-region has exhibited notable improvements. In contrast, the LZB and CBS sub-regions have consistently maintained a favorable marine ecological environment. These results indicate a correlation between the state of the marine ecological environment in each sub-region and the level of development of the adjacent urban centers. Additionally, the assessment of the marine ecological environment presented here further endorses the practicability and rationality of using the Chl-*a* result with RBF-Linear interpolation as a suitable evaluation method for the marine ecological environment. Meanwhile, this will provide a new technical method for the research of Chl-*a* in the future.

## Conclusion

The present study capitalized on an extensive dataset of in-situ Chl-*a* measurements encompassing the Bohai Sea. The observational data was collected across multiple seasons, including the months of March, May, August, and October, over a period of two years, from 2016 to 2018. The number of observational stations for each month consistently exceeded 300, which provided abundant data for undertaking further research.

To compare the efficacy of different three-dimensional spatial interpolation methods, we utilized the Chl-*a* concentration data collected in August 2017 from the Bohai Sea. Five methods, namely RBF-Linear, RBF-Gaussian, RBF-Multi quadrics, IDW, and Kriging were compared by employing tenfold cross-validation, correlation analysis, and assessing spatial distribution of Chl-*a* concentration. Our results indicate that the RBF-Linear interpolation method displays the lowest error and highest consistency with the in-situ data, along with the most logical spatial distribution, making it the most effective interpolation method for the study. Subsequently, we utilized this approach to obtain the complete three-dimensional spatial distribution of Chl-a concentration in the Bohai Sea for the months of March, May, August, and October from 2016 to 2018.

This study further utilized the results of the Chl-*a* interpolation to investigate the spatial and temporal distribution of Chl-*a* concentration in the Bohai Sea from 2016 to 2018, along with the marine ecological environment during this time period. The investigation revealed that the high concentration of Chl-*a* was primarily present in the coastal surface water, especially near the estuaries and mariculture areas, with March and August exhibiting the most significant peaks. To assess and compare the marine ecological environment across the four sub-regions of the Bohai Sea, we employed two crucial indicators, i.e., the total Chl-*a* concentration and the area with high Chl-*a* concentration. The analysis indicated that the marine ecological environment in BHB was the most concerning since it exhibited a declining trend during the early spring of 2016 to 2018, with only modest improvement in following seasons. The LDB sub-region showed significant improvement, with a dramatic improvement observed between 2016 and 2018. In contrast, the LZB and CBS sub-regions had consistently maintained a healthy coastal environment over the years. At the same time, the preceding analysis demonstrates that the three-dimensional space field of Chl-*a* obtained by the RBF-Linear interpolation method can be directly used in the investigation and analysis of Chl-*a*, with reasonable results.

In summation, the RBF-Linear technique is utilized to interpolate Chl-*a* concentration in three-dimensional space, and high-quality reconstructed images are obtained that capture significant spatio-temporal changes. Our research demonstrates that RBF-Linear is preferable to other interpolation techniques and can be directly applied to the spatial–temporal analysis of Chl-*a* and the evaluation of the marine ecological environment. In addition, it can be utilized to evaluate satellite products and enhance the precision of ecological model output in future research.

## Data Availability

For data acquisition, please consult corresponding author Shufang Zhang, author email address: sfzhang@nmemc.org.cn.
