# Author Correction: Three-dimensional spatial interpolation for chlorophyll-*a* and its application in the Bohai Sea

**DOI:** 10.1038/s41598-023-37864-w

**Published:** 2023-06-30

**Authors:** Zihan Zhao, Rushui Xiao, Junting Guo, Yuming Zhang, Shufang Zhang, Xianqing Lv, Honghua Shi

**Affiliations:** 1 Frontier Science Center for Deep Ocean Multispheres and Earth System (FDOMES) and Physical Oceanography Laboratory, Ocean University of China, Qingdao, China; 2 South China Institute of Environmental Sciences, Ministry of Ecology and Environment, Guangzhou, 510530 China; 3 National Marine Environment Monitoring Center, Dalian, 116023 China; 4 First Institute of Oceanography, Ministry of Natural Resources, Qingdao, 266061 China; 5 Laoshan Laboratory, Qingdao, 266237 China

Correction to: *Scientific Reports* https://doi.org/10.1038/s41598-023-35123-6, published online 16 May 2023

The original version of this Article contained an error in Affiliation 5, which was incorrectly given as ‘Qingdao National Laboratory for Marine Science and Technology, Qingdao, 266237, China.’

The correct affiliation is listed below:

Laoshan Laboratory, Qingdao, 266237, China

The original Article has been corrected.

